# The design, evaluation, and reporting on non‐pharmacological, cognition‐oriented treatments for older adults: Results of a survey of experts

**DOI:** 10.1002/trc2.12024

**Published:** 2020-06-07

**Authors:** Sharon Sanz Simon, Mary Castellani, Sylvie Belleville, Tzvi Dwolatzky, Benjamin M. Hampstead, Alex Bahar‐Fuchs

**Affiliations:** ^1^ Cognitive Neuroscience Division Department of Neurology Columbia University New York New York; ^2^ Old Age Research Group (PROTER) Department of Psychiatry São Paulo Medical School University of São Paulo São Paulo Brazil; ^3^ Academic Unit for Psychiatry of Old Age Department of Psychiatry University of Melbourne Melbourne Australia; ^4^ Psychology Department Université de Montréal Montreal Canada; ^5^ Research Center Institut Universitaire de Gériatrie de Montréal Montreal Canada; ^6^ Rambam Health Care Campus and Rappaport Faculty of Medicine Technion—Israel Institute of Technology Haifa Israel; ^7^ Mental Health Service VA Ann Arbor Healthcare System Ann Arbor Michigan; ^8^ Neuropsychology Section Department of Psychiatry University of Michigan Ann Arbor Michigan

**Keywords:** aging, Alzheimer's disease, cognitive intervention, cognitive rehabilitation, cognitive stimulation, cognitive training, dementia, mild cognitive impairment

## Abstract

**Introduction:**

Cognitive decline and dementia significantly affect independence and quality of life in older adults; therefore, it is critical to identify effective cognition‐oriented treatments (COTs; eg, cognitive training, rehabilitation) that can help maintain or enhance cognitive functioning in older adults, as well as reduce dementia risk or alleviate symptoms associated with pathological processes.

**Methods:**

The Cognitive Intervention Design Evaluation and Reporting (CIDER), a working group from the Non‐Pharmacological Interventions Professional Interest Area (NPI‐PIA) of the Alzheimer's Association conducted as survey in 2017 with experts in COTs worldwide. The survey's aims were three‐fold: (1) determine the common attitudes, beliefs, and practices of experts involved in the COTs research targeting older people; (2) identify areas of relative agreement and disagreement among experts in the field; and (3) offer a critical review of the literature, including recommendations for future research.

**Results:**

The survey identified several areas of agreements among experts on critical features of COTs, and on study design and outcome measures. Nevertheless, there were some areas with relative disagreement. Critically, expert opinions were not always supported by scientific evidence, suggesting that methodologic improvements are needed regarding design, implementation, and reporting of COTs. There was a clear consensus that COTs provide benefits and should be offered to cognitively unimpaired older adults, mild cognitive impairment (MCI), and mild dementia, but opinions differed for moderate and severe dementia. In addition, there is no consensus on the potential role of COTs in dementia prevention, indicating that future research should prioritize this aspect.

**Discussion:**

Evidence of COTs in older adults is encouraging, but additional evidence is needed to enhance dementia prevention. Consensus building and guidelines in the field are critical to improve and accelerate the development of high‐quality evidence for COTs in cognitively unimpaired older adults, and those with MCI and dementia.

## 1 INTRODUCTION

There is considerable growth of interest in non‐pharmacological interventions for older people due to the potential of such interventions in reducing dementia risk and alleviating symptoms associated with age‐related pathological processes. Cognition‐oriented treatments (COTs) represent a group of non‐pharmacological intervention approaches that has received a great amount of attention from both the public and scientific community, and this is reflected in their inclusion in the World Health Organization's guidelines for risk reduction of cognitive decline and dementia.^1^ The term COTs refer to a range of techniques applied to engage cognition with various degrees of breadth and specificity. These techniques aim to improve or maintain cognitive processes, and/or to address the impact of cognitive impairment on functional ability in daily life.^2^ COTs are increasingly being recognized as beneficial for older people, since engaging in cognitively stimulating activities can be protective for age‐related cognitive decline^3^ and dementia,^4^, ^5^ possibly by increasing cognitive reserve and resilience in later life.^6^, ^7^

Several terms have been used to refer to the methodologies adopted by COTs, and these include cognitive stimulation, cognitive training, and cognitive rehabilitation. Whereas *cognitive stimulation* involves activities targeting cognitive or social functioning in a non‐specific manner, *cognitive training* tends to be more specific, and applies or teaches theoretically oriented techniques that target cognitive processes. *Cognitive rehabilitation* involves programs tailored for individual goals, and is centered on performance of specific activities of daily living.^8^ A further distinction described in the literature is between rehearsal‐based COT approaches, which emphasize the repetition of information over time, and assumes that cognitive processes will improve through repeated practice; and strategic approaches, which emphasize altering the manner in which information is processed or a task is performed to compensate for cognitive deficits.^9^ Strategic approaches can involve the use of *external aids* to facilitate task performance (eg, using a calendar, grocery list, or note system), or of *internal strategies*, reflecting cognitive “*tools*” that facilitate a deeper level of processing and task performance (eg, mental imagery, mnemonics to facilitate organization, and association of new information).^10^

One of the premises of COTs (especially those involving a cognitive training component) is that training or teaching techniques to improve a cognitive ability or process will lead to transfer of gains beyond the immediate context of the intervention. Researchers typically discuss transfer in terms of near versus far transfer, but the field lacks a consensus/precise definition of what “near” and “far” in fact constitute. The inconsistency in the way near and far transfers are conceptualized and operationalized reflect in part a difficulty in identifying the most meaningful way to draw boundaries between “near” and “far,” which leads to a limited understanding of transfer effects in the COT literature and restricts our ability to compare the conclusions across studies. According to Karbach and Verhaeghen, near transfer is demonstrated by improvement in performance on tasks not explicitly trained, but that measure the same construct as the construct trained, whereas far‐transfer is demonstrated by improvement in performance on tasks measuring a different construct than the one trained.^11^ A further proposed possibility is to distinguish between *content‐*based transfer, reflecting transfer of gains from trained tasks to untrained tasks of a similar nature, and content, and *context‐*based transfer, which reflects transfer to situations different from the training context in content and format, such as everyday activities.^12^ To date, it appears that for the most part, the literature has referred mainly to transfer in terms of “near” versus “far,”^13^ even though a major challenge for the field remains the demonstration of transfer of benefits from COTs to different contexts and meaningful activities of daily living.

The body of evidence in relation to COTs across the aging spectrum has been summarized recently in a Systematic Overview (Gavelin et al.),^14^ indicating efficacy of COTs in improving cognitive performance in older adults, despite the scarcity of high‐quality evidence and heteroneity in reported findings. There is evidence to suggest the possible benefits of COTs for global cognition in cognitively unimpaired (CU) older adults,^11^, ^15^, ^16^, ^17^, ^18^, ^19^, ^20^, ^21^, ^22^, ^23^ in those with mild cognitive impairment (MCI),^10^, ^22^, ^24^, ^25^, ^26^, ^27^, ^28^, ^29^, ^30^ and, to some extent, in people with dementia (PwD).^2^, ^26^, ^31^, ^32^, ^33^, ^34^, ^35^, ^36^

In the context of CU older adults, randomized controlled trials have shown benefits of COTs in cognitive domains that typically decline as a function of age, such as attention and executive control,^9^, ^13^, ^37^, ^38^, ^39^, ^40^ working memory,^41^, ^42^, ^43^, ^44^ speed of processing,^45^, ^46^ episodic memory, and reasoning.^45^, ^47^ The combination of different domains (ie, executive processes, working memory, episodic memory, and speed) and other modalities of lifestyle intervention (ie, exercise, nutritional counseling, and health management) has also resulted in cognitive gains.^48^ In addition, there is evidence that domain‐specific cognitive‐training protocols can lead to cognitive and functional benefits lasting as long as 10 years,^3^ and may be associated with reduced dementia risk.^5^ Other studies have shown memory benefits after internal cognitive strategy training (eg, mnemonics) for CU older people and those with MCI,^24^, ^49^, ^50^, ^51^, ^52^, ^53^, ^54^, ^55^ as well as following external strategy training, such as the use of a calendar or note system.^56^, ^57^ Cognitive strategy training focused on memory has been the main approach studied in MCI, since memory deficits are prevalent in this population, and despite encouraging results, there are also negative findings in the primary cognitive outcomes.^58^, ^59^ Beyond cognition, there is some evidence that COTs may lead to gains in quality of life, mood, self‐efficacy,^60^ and metacognition in those with MCI.^24^, ^54^, ^61^ Considering PwD, a recent systematic review concluded that, relative to a control intervention, cognitive training may be associated with moderate effects on overall cognition, but the cognitive gains were no different than gains associated with alternative structured treatments.^2^ Data from individual trials suggest that goal‐oriented cognitive rehabilitation may be an effective approach to improve personal satisfaction with the performance of relevant activities of daily living,^31^, ^33^, ^34^, ^62^ and a protocol for a systematic review of the literature in this area has been published recently (Kudlicka et al.).^63^

**RESEARCH IN CONTEXT**

1. Systematic review: The authors described the beliefs, attitudes, and practices of experts in cognitive‐oriented treatments (COTs) in older population, and compared these with the evidence in the field. The survey identified several areas of agreement among experts on critical features of COTs, study design, and outcome measures. Nevertheless, there were some areas with relative disagreement. Critically, opinions were not always supported by scientific evidence.
2. Interpretation: Despite the encouraging results of COTs in older adults, there are inconsistent results in the field that limit the quality of evidence. The findings indicate that methodological improvements in design, implementation, and reporting on COTs is a priority in order to enhance evidence based‐practice, dementia prevention, and public health recommendations.
3. Future directions: The manuscript proposes that future COTs research should provide more evidence on dementia prevention. In addition, it is proposed that guidelines for COTs research should be developed, in order to accelerate the development of high‐quality evidence of COTs to cognitively unimpaired older adults, those with MCI, and with dementia.

Despite the encouraging evidence of COT‐related benefits in the older population, it is not clear to what extent clinicians and researchers rely on these interventions, and what their opinion is regarding their use. Methodological issues in the design, evaluation, and reporting of COT trials continue to challenge researchers, and clinicians, leading to difficulty drawing firm and consistent conclusions, and more than likely restricting their implementation in clinical and community settings. These include substantial variability across intervention design and trial methods, typical small sample sizes and limited statistical power, which lead to inconsistencies across studies. In addition, intervention protocols vary in terms of the cognitive processes targeted, the approach utilized (eg, rehearsal or strategy‐based), as well as such factors as the setting, format, level of supervision, frequency, dose, type of control condition (if any), outcome measures, follow‐up period (if any), and statistical methods used. In particular, the rationale behind many methodological decisions is often unclear and/or unspecific, and testable hypotheses are not always provided.

Against this background, we created the Cognitive Intervention Design Evaluation and Reporting (CIDER) group in 2014.^64^, ^65^ The CIDER group is an international expert working party that aims to advance methodological rigor in COT trials, encourage greater consensus and collaboration in the field, and promote more responsible dissemination of research evidence. The group comprises academics and clinicians involved in the research and delivery of COTs to older adults. CIDER is committed to advancing evidence‐based research and practice in this area (including by establishing a novel evidence synthesis platform (www.cogtale.org),^66^ and to this end is working on the development of research guidelines, and possibly, future consumer guidelines. In 2017, CIDER conducted a survey of experts (researchers and clinicians) to gain insight as to their attitudes, beliefs, and practices in relation to several topics involving COTs in older adults (specific details are outlined later). The goal of the survey was to identify areas of relative agreement and disagreement, and to clarify beliefs and gaps in knowledge of experts regarding the design and implementation of COTs. The findings of this survey would potentially form the basis for ongoing expert consensus‐building activity, while keeping in mind that COT‐related research and practice around the world is likely to reflect beliefs and values in addition to scientific evidence. It should be a priority to identify relevant factors that influence COT research in order to improve the design, implementation, and reporting of study methods. Greater consensus among researchers and clinicians is certainly likely to improve and accelerate the development of evidence‐based practice.

### 1.1 The present study

The present report details the results of the survey of experts. The primary aim of the survey was to determine the common attitudes, beliefs, and practices of researchers involved in the design and conduct of COT studies targeting older people. A second aim was to identify areas of relative agreement and disagreement among experienced researchers and clinicians involved in COT research and implementation. A third aim was to offer a critical review of the literature and propose recommendations for research methodology based on the survey responses. It was anticipated that the survey would reveal gaps in knowledge and beliefs of experts regarding the design and implementation of cognitive intervention studies, including aspects such as characteristics of COTs.

## 2 METHOD

### 2.1 Survey

The survey was developed in an iterative fashion by collaboration between CIDER members in early 2017 and was designed to take ≈30 minutes to compete. It aimed to investigate the knowledge, common beliefs, and attitudes regarding COTs held by researchers and clinicians who work with older people. The survey was divided into eight sections, as presented in Table 1.

**TABLE 1:** Summary of survey sections

Survey topic	General content^a^
1. Respondent characteristics	Background information, demographics (ie, age, gender, country), category of professional training, and professional experience of the experts.
2. Features and components	Relevance of cognitive focus (eg, multiple cognitive domains or in isolation) and approaches, how to incorporate strategies, and what are the component priorities for effectiveness.
3. Target population	Specificities of each population targeted in COTs for older adults––CU, MCI, or dementia—and likelihood of each to benefit from different COTs.
4. Settings and mode of delivery	Importance of type of settings (eg, clinical, home, community, combined), format (eg, group, individual, combined) and level of supervision for effectiveness.
5. Dose, frequency, and duration	Relevance of number of sessions, intensity per week, trials, and minutes engaged in a session, total duration in short‐ and long‐term effects, and role of booster sessions for maintenance.
6. Outcomes and assessments	How to measure relevant outcomes, types of cognitive measures/assessments, self‐report measures, and priorities when considering a relevant outcome for effectiveness.
7. Evaluation of treatment efficacy	Ways to demonstrate COT efficacy, control group conditions (eg, active, “placebo”, waitlist, treatment as usual), between‐intervention design, level of evidence.
8. Prescription of COTs	Agreement on whether the evidence is strong enough to prescribe particular COT to specific populations.

a Questions considered the specificity of each population: cognitively unimpaired (CU), mild cognitive impairment (MCI), and dementia.

### 2.2 Participants

Potential participants were recruited via the Non‐Pharmacological Interventions Professional Interest Area (NPI‐PIA) of Alzheimer's Association ISTAART (International Society to Advance Alzheimer's Research and Treatment) network, of which CIDER is a working party, as well as by directly contacting the first and last author of COT trials published in recent years. The survey was sent to 120 academic and/or clinical researchers with expertise in the design and delivery of cognition‐focused interventions for older adults. The experts’ work was focused on at least one of three target populations: (1) healthy older adults (eg, CU), (2) older adults at significant risk for dementia (eg, MCI), or (3) adults living with dementia (eg, verified dementia diagnosis).

### 2.3 Procedure

Preliminary concepts and ideas for the survey were discussed during regular monthly CIDER meetings in 2016, and the survey was drafted in an iterative fashion in early 2017. The project was reviewed and approved as a Negligible Risk Research Project by the Melbourne Health Human Ethics Review Committee (Melbourne, Australia, approval number QA2017037). The survey was developed and disseminated using the Qualtrics online survey tool (Qualtrics, Provo, UT, USA), and was available from May to June 2017. In addition to being sent directly to specific researchers based on their scholarly output, the survey was further promoted via the international NPI‐PIA network, and the Alzheimer's Association ISTAART newsletter. Participant information was provided in a preamble at the start of the online survey. Respondents were then asked to give consent in order to access the survey, which could be discontinued at any time by the respondent closing their browser.

### 2.4 Consensus meeting and discussion

After the survey was completed, the CIDER committee organized the presentation of the survey results at a consensus‐building meeting at the Alzheimer's Association International Conference (AAIC) in London, UK during July 2017. The meeting included nine leading authors in the field from different countries (ie, Australia, Canada, China, Israel, United Kingdom, and United States). The participants were encouraged to actively discuss the survey topics, provide their opinions, and raise relevant hypotheses. The objective of this meeting was to present and discuss the survey results, and the level of agreement on the effectiveness of COTs for older adults who were CU, or were determined to have MCI or dementia.

### 2.5 Data analysis

The data were analyzed by quantifying frequencies associated with different response options to the various survey questions. Responses are reported in terms of the level of agreement with Likert scales, ranking of items from high to low, and the frequency of selected options from a list. Quantities are reported as percentages, with 100% representing all completed survey responses recorded.

## 3 RESULTS

### 3.1 Respondent characteristics

**FIGURE 1:**
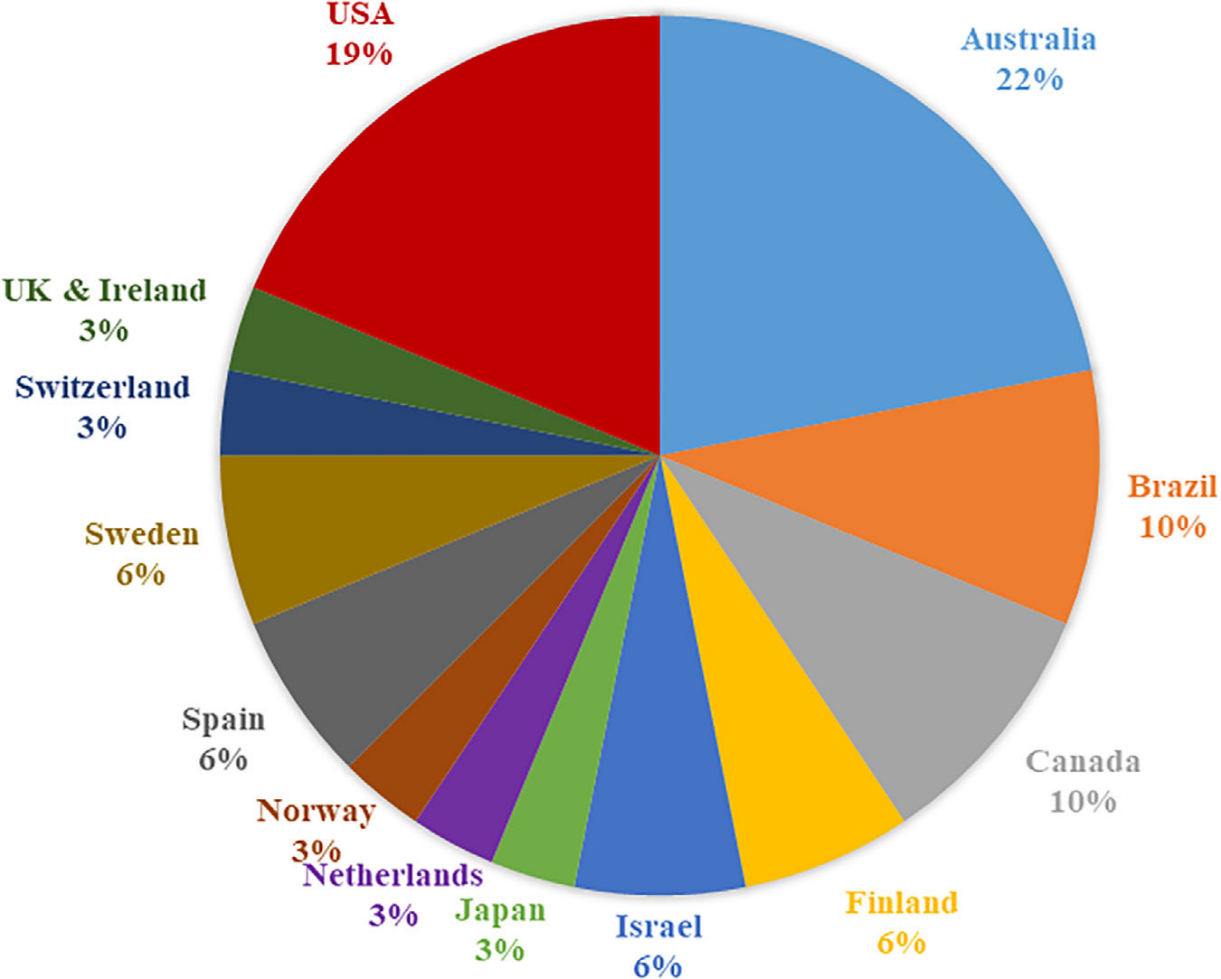
Countries of survey experts

Of the experts invited by email, 39 (32%) commenced and 32 (26.5%, 50% women) completed the survey, with respondents from 15 countries (Figure 1). Age range varied from 25 to 75 years, and 81% had over 10 years of experience working in medical (25%), psychological (75%), academic (60%), or combined clinical and research (40%) settings. All respondents reported involvement with implementation of cognitive interventions, and 22% were able to prescribe medications. Approximately one third of the respondents had expertise in COTs with MCI (33%), followed by mild‐to‐moderate dementia (25%), CU (23%), moderate‐to‐severe dementia (8%), and other populations (eg, Parkinson disease, late‐life depression, subjective cognitive decline, semantic dementia). Regarding intervention type, 41% of the respondents reported expertise in cognitive training, 21% in a mix of methods, 15% in strategy‐oriented techniques, 12% in cognitive stimulation, 3% in cognitive rehabilitation, and 3% in education programs. For illustration of expert characteristics, see Figure S1.

### 3.2 COT critical features

Respondents were asked to indicate their opinion regarding the relevance of several intervention components and classify them as: (1) “Critical”, meaning that the treatment is very unlikely to be effective without this component; (2) “Optional”, meaning that the treatment may be improved by the inclusion of this component, but the success does not depend on it; or (3) “Irrelevant”, meaning that the treatment feature is unlikely to be associated with any benefits.

**FIGURE 2:**
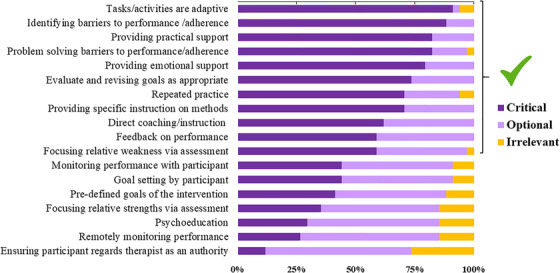
COT treatment features

COT features are summarized in Figure 2. There was relative consensus among the respondents on the features deemed *critical* to the effectiveness of COTs, since >50% of the responders considered the following features as “critical”: (1) tasks or activities should be adaptive; (2) barriers to performance and adherence should be identified; (3) problem solving should be provided for barriers to performance and adherence; (4) practical and (5) emotional support should be available to participants; (6) goals should be evaluated and revised as appropriate; (7) repeated practice; (8) specific instruction on intervention methods should be provided; (9) direct coaching or instructions; (10) feedback on performance; and (11) focus on relative weaknesses. There was also relative agreement that certain intervention features should be considered or incorporated *optionally* in COT trials, including (1) focusing on relative strengths of the participants; (2) psychoeducation; (3) remote performance monitoring; and (4) ensuring that the participant regards therapist as an authority. Finally, there was relatively low consensus regarding the relevance of some intervention features such as: (1) monitoring performance with participants; (2) goal setting by the participant; and (3) setting pre‐determined intervention goals.

### 3.3 COT approaches and targets

**FIGURE 3:**
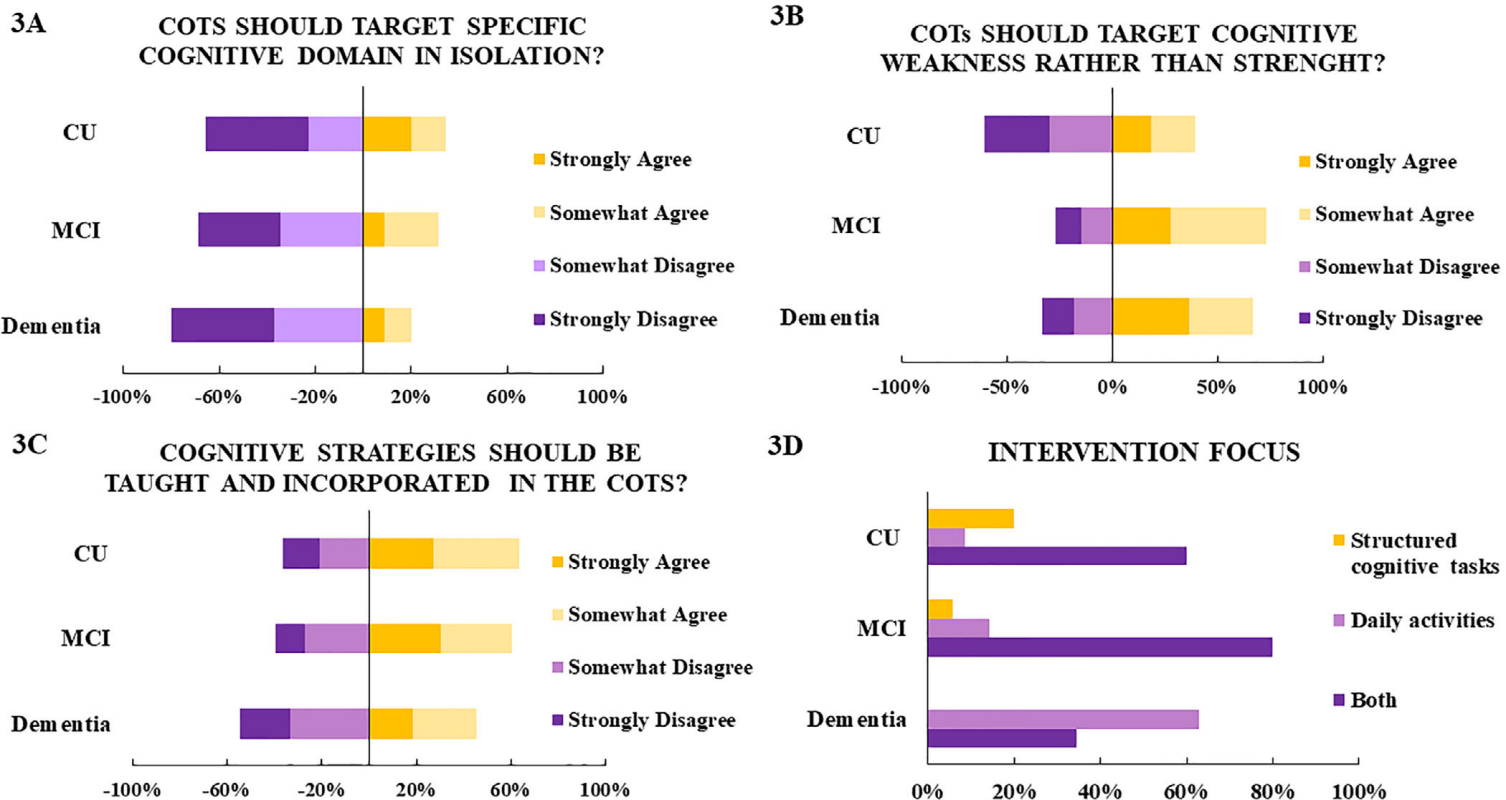
COT approaches and targets

The majority of respondents (80%) agreed that the distinction between COT approaches typically described in the literature (eg, cognitive stimulation, cognitive training, and rehabilitation) reflect important differences in treatment and mechanisms of action. For example, 70.5% of respondents considered individualized goal setting (GS) as an essential component of the cognitive rehabilitation approach only, but not for cognitive training (14.7%), strategy training (8.8%), and cognitive stimulation (5.8%). In relation to the populations being targeted by a particular type of intervention, there was relative agreement that COTs should not target a single cognitive domain, particularly for individuals with mild‐moderate dementia (80% agreement), but also for older adults with MCI and CU (68.5% and 65.7% of agreement, respectively) (see Figure 3A). According to most respondents, COTs should focus on impaired or weaker cognitive functions (ie, cognitive weakness) rather than intact cognitive functions (ie, relative cognitive strength), particularly in those with MCI (72.7%), and dementia (66.6%), but this was not deemed as important in CU older adults (39.3%). It was agreed that cognitive strategies were more relevant to outcomes for CU older adults (63.6%) and those with MCI (60.6%), but not so useful for PwD (45.4%) (Figure 3C). Regardless of the intervention approach or the targeted cognitive domains, 67.6% of the respondents agreed that cognitive strategy training is the primary mechanism of action required to support transfer of gains from trained to untrained tasks. Regarding focus of the intervention (Figure 3D), most of the respondents believed that both structured cognitive tasks and daily activities should be targeted in COTs for CU adults (60% agreement), and MCI (80%). However, for PwD, the focus of the intervention considered optimal was daily activities only (62%).

### 3.4 COT design and outcome measures

**FIGURE 4:**
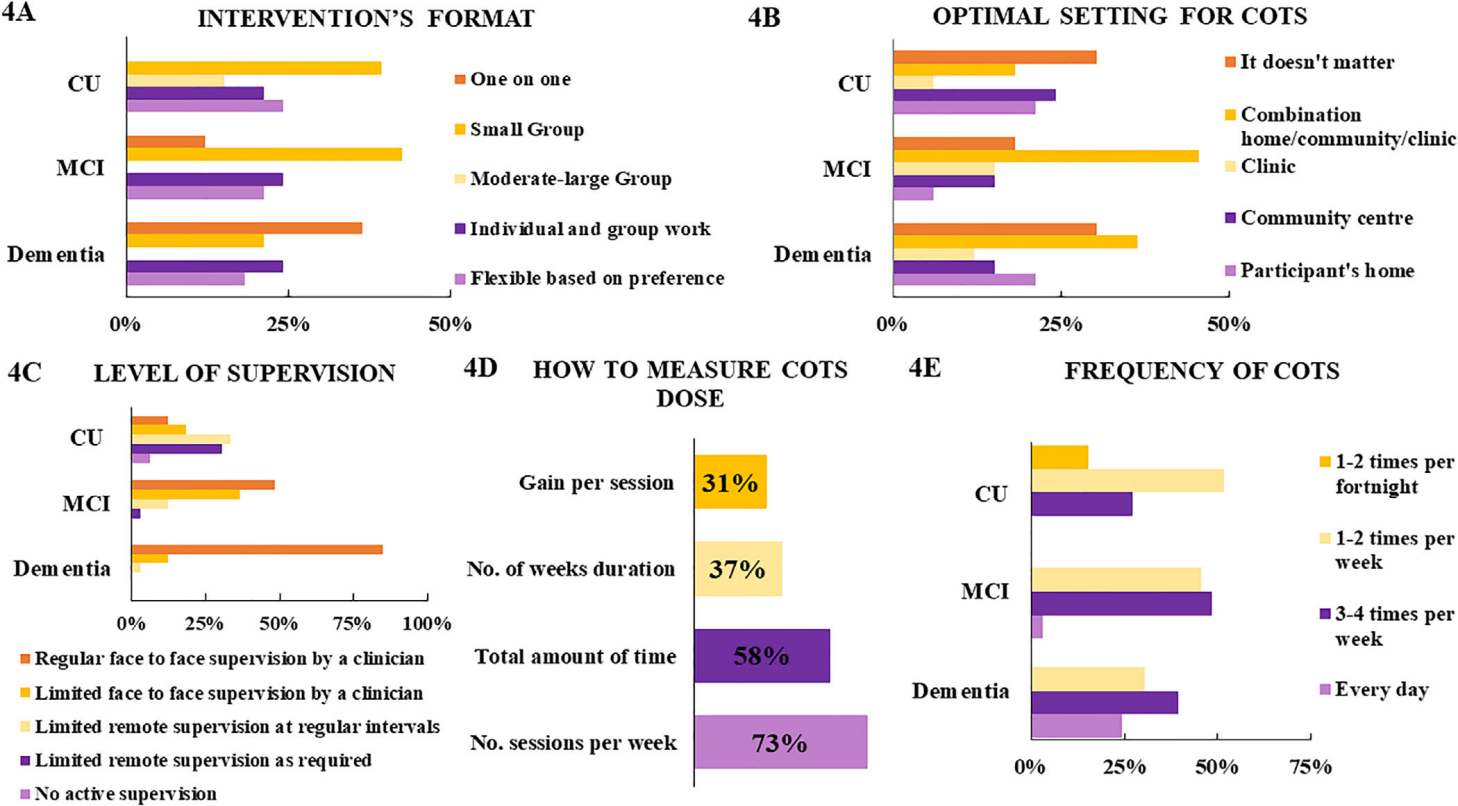
COT design

The design characteristics of COTs were analyzed considering five categories: format of intervention (eg, individual or group delivery), setting (eg, clinic or community location), level of supervision (eg, remote or face to face), dose (eg, total number of sessions), and frequency (eg, how many sessions per week or month) (Figure 4). Regarding the intervention format (Figure 4A), the preferred format for both CU older adults and MCI was *small group* (39.3%, and 42.2%, respectively); and for those with mild to moderate dementia was *one on one* (36.3%). In addition, respondents agreed that *moderate‐large group* is not optimal for those with MCI or dementia, nor a *one on one* format for CU older adults. When respondents were asked to rank the optimal setting for COTs (Figure 4B), the dominant view concerning people with MCI and dementia was the *combination of home, community, and clinic*, while respondents most commonly stated that the setting *does not matter* in relation to CU older people. In relation to level of supervision (Figure 4C), for CU people there was relative agreement (63.6%) that *limited remote supervision* (as required or at irregular intervals) would be optimal, whereas for MCI and dementia, *face‐to‐face supervision by a clinician* would be more appropriate (84% of agreement for MCI, and 97% for dementia). Nevertheless, for MCI, *face‐to‐face supervision* could be *limited* or *regular*, while it should be mostly *regular* in the context of dementia. Regarding dose (Figure 4D), respondents were asked to rank in order of usefulness different dose measurement approaches when designing a treatment trial. The main approaches were *number of sessions per week* and *total amount of time*. Specifically, 42.3% considered *number of sessions per week* the most useful dose criterion, and 73% ranked it as the first or second most useful classification, whereas 26.9% believed that *total amount of time* was the more relevant dose classification, and 58% ranked it as the first or second most useful criterion.

The respondents were asked to choose the minimum frequency deemed to confer cognitive or functional benefits within different populations (Figure 4E). For CU older adults, a marginal majority of respondents (51.5%) considered the minimum COTs frequency to be *1 to 2 times per week*; however, 27.2% considered the minimum frequency to be *3 to 4 times per week*, and 15.1% reported *1 to 2 times per fortnight* to be the minimum frequency. For people with MCI, respondents were divided; 48.4% considered *3 to 4 times per week* as the minimum frequency, whereas 45.4% considered *1 to 2 times per week*. Regarding PwD, opinions were also divided, 39.3% of the respondents considered a minimum frequency of *3 to 4 times per week*, 30.3% considered *1 to 2 times per week*, and 24.2% reported that COTs should be delivered *daily*.

**FIGURE 5:**
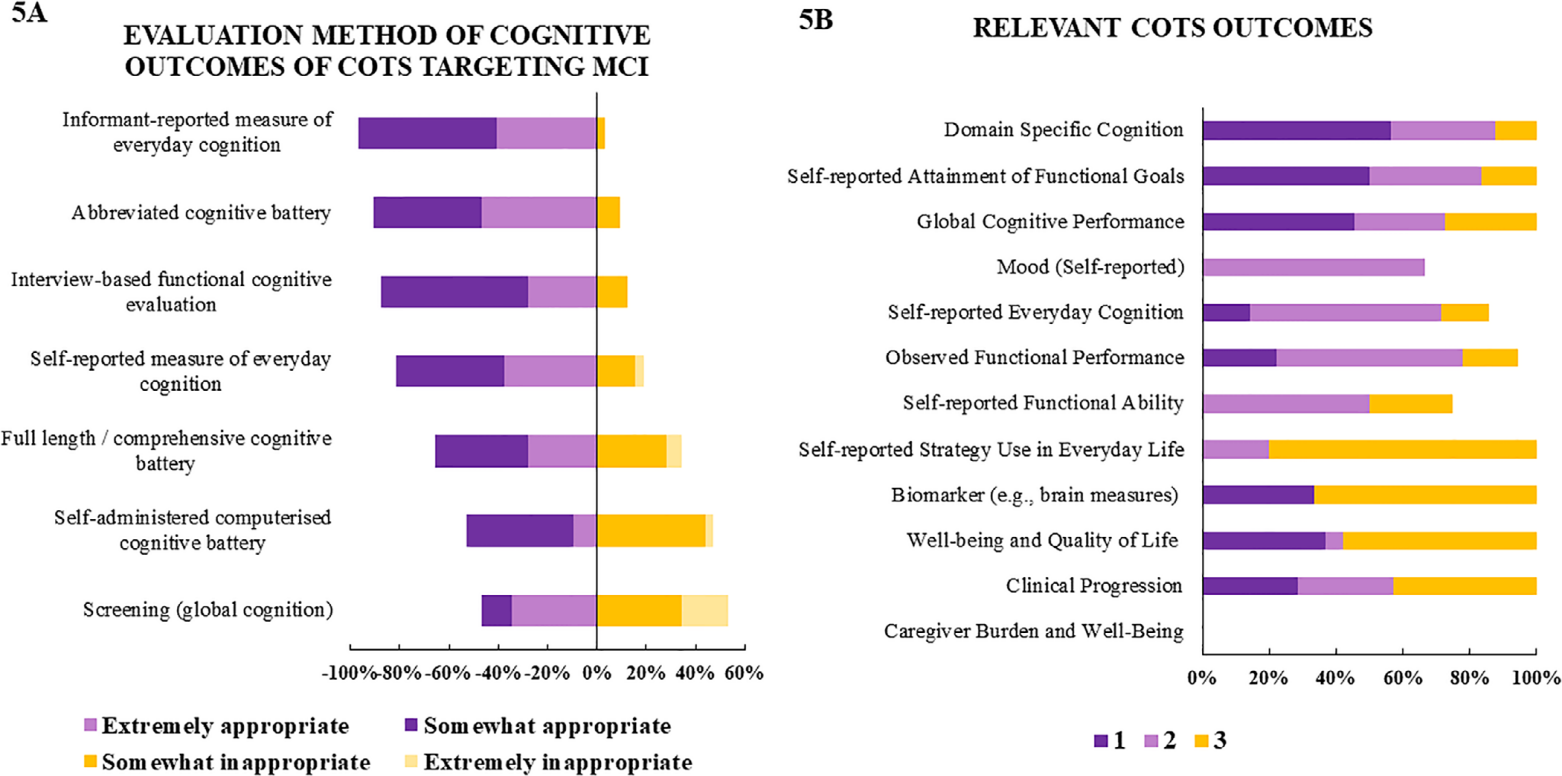
COT outcome measures

Participants were asked to rate methods of evaluation of cognitive outcomes in COTs targeting people with MCI (Figure 5A), while considering issues of time, resources, and participant burden. The main evaluation methods considered appropriate were *informant‐reported measure of everyday cognition* (96.8% agreement) and *abbreviated cognitive battery* (90.6%), followed by *interview‐based functional cognitive evaluation* (87.5%), *self‐measure of everyday cognition* (81.2%), and *full length/comprehensive cognitive battery*(65.6%). The evaluation methods with more disagreement was self‐administered *computerized cognitive battery* (53.1%), and *screening battery* (global cognition) (46.8%). Moreover, respondents ranked the three most relevant outcomes among a list of 12 outcomes, regardless of the population (Figure 5B). The COTs outcomes ranked as first in term of relevance were *domain specific cognition* (56.2%), *self‐reported attainment of functional goals* (50%), and *global cognitive performance* (45.4%). The outcomes ranked as second most important were *mood* (66.5%), *self‐reported everyday cognitio* *n*(57.1%), *observed functional performance* (55.5%), and *self‐reported functional ability* (50%). Last, ranked as third most important outcome were *self‐reported strategy use in everyday life* (80%), *biomarkers* (eg, brain measures) (66.6%), *well‐being and quality of life* (57.8%), and *clinical progression* (42.8%).

### 3.5 Population benefit, prescription, and dementia prevention

**FIGURE 6:**
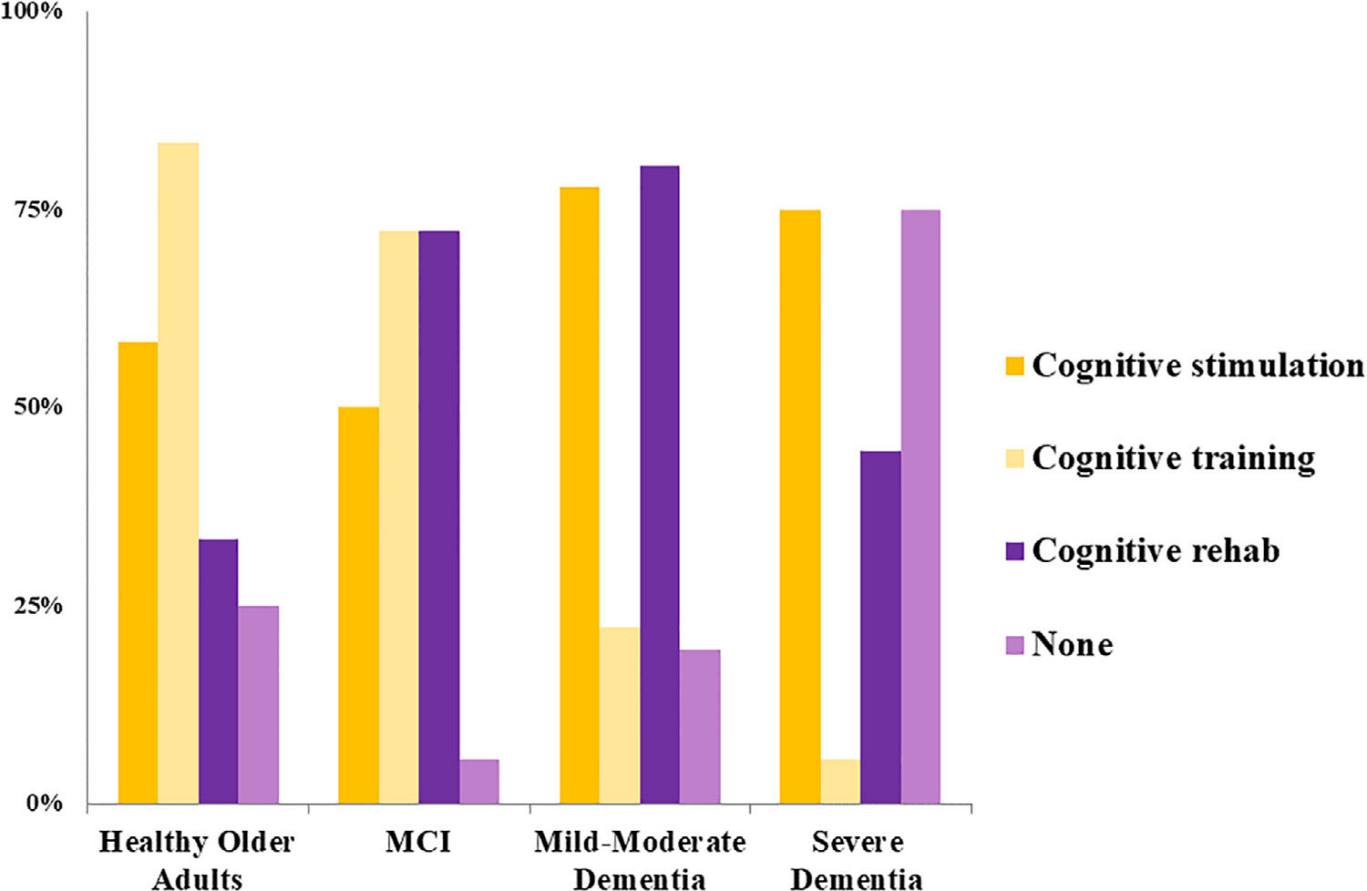
Most useful COT approach for each population

According to respondents, the population most likely to benefit from COTs is people with MCI (95%), followed by CU older people (69%), and finally people with mild dementia (30%). People with moderate (6%) and severe (0%) dementia were not considered likely to benefit from COTs. In addition, respondents selected different intervention approaches as more useful to specific target groups (Figure 6). For CU older adults, *cognitive training* was considered the most relevant approach, whereas for people with MCI, both *training and rehabilitation* were considered equally useful. For people with mild‐to‐moderate dementia, *cognitive stimulation and rehabilitation* were considered the best approaches. For severe dementia, respondents preferred either *cognitive stimulation or no intervention* in equal proportion, indicating a lack of consensus in this area.

**FIGURE 7:**
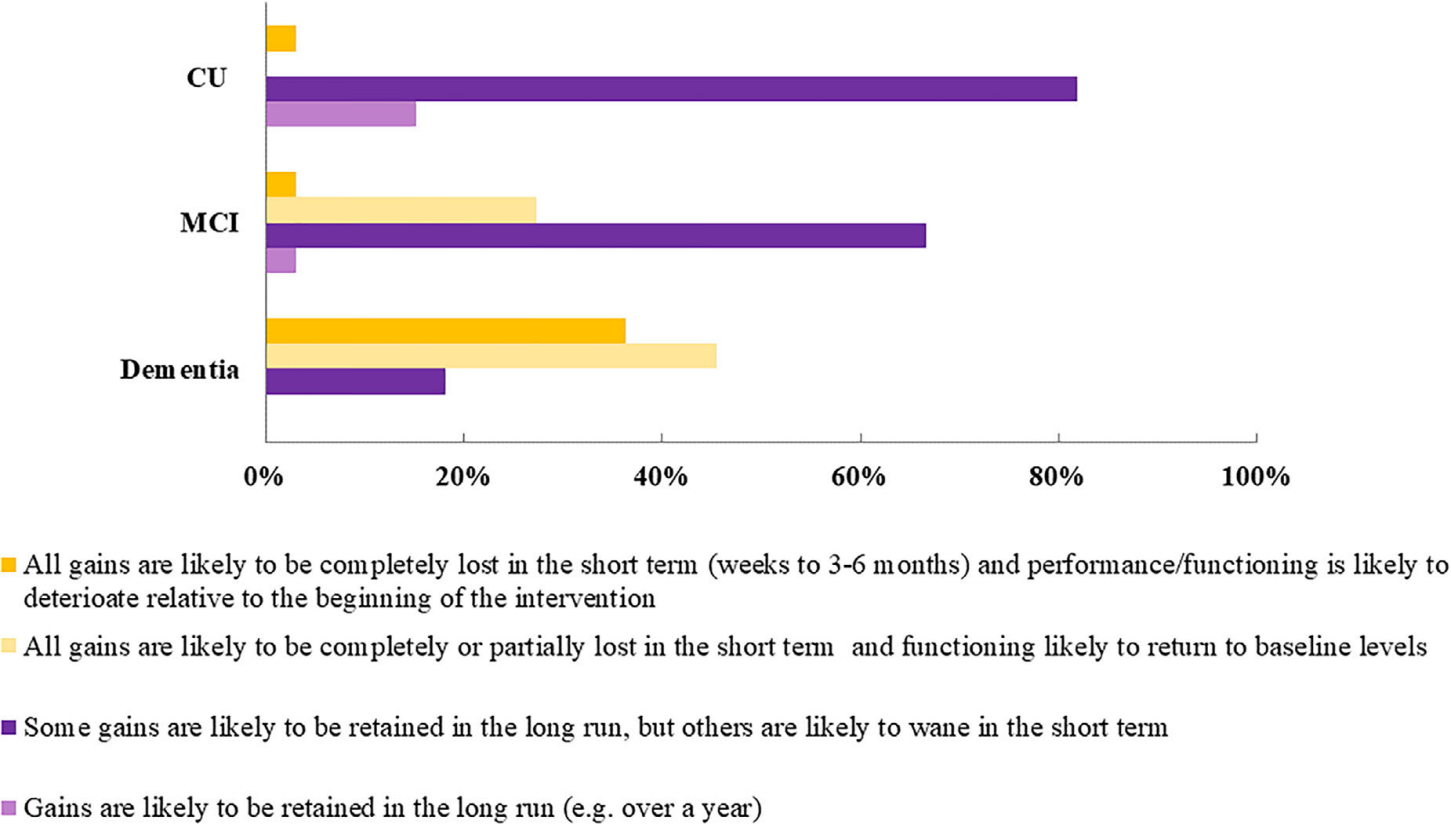
Maintenance of COT benefits

Regarding maintenance of benefits (Figure 7), most respondents believed that for both CU older adults (81.8%) and people with MCI (66.6%), some gains would be retained in the *long term* (ie, over a year), but others are likely to wane in the *short term* (3 to 6 weeks). For PwD, 45% of respondents considered that all gains are likely to be *completely or partially lost* in the short term and that functioning is likely to return to baseline levels; and 36% considered that gains would *completely be lost* in the short term and functioning is likely to deteriorate relative to the beginning of the intervention.

**FIGURE 8:**
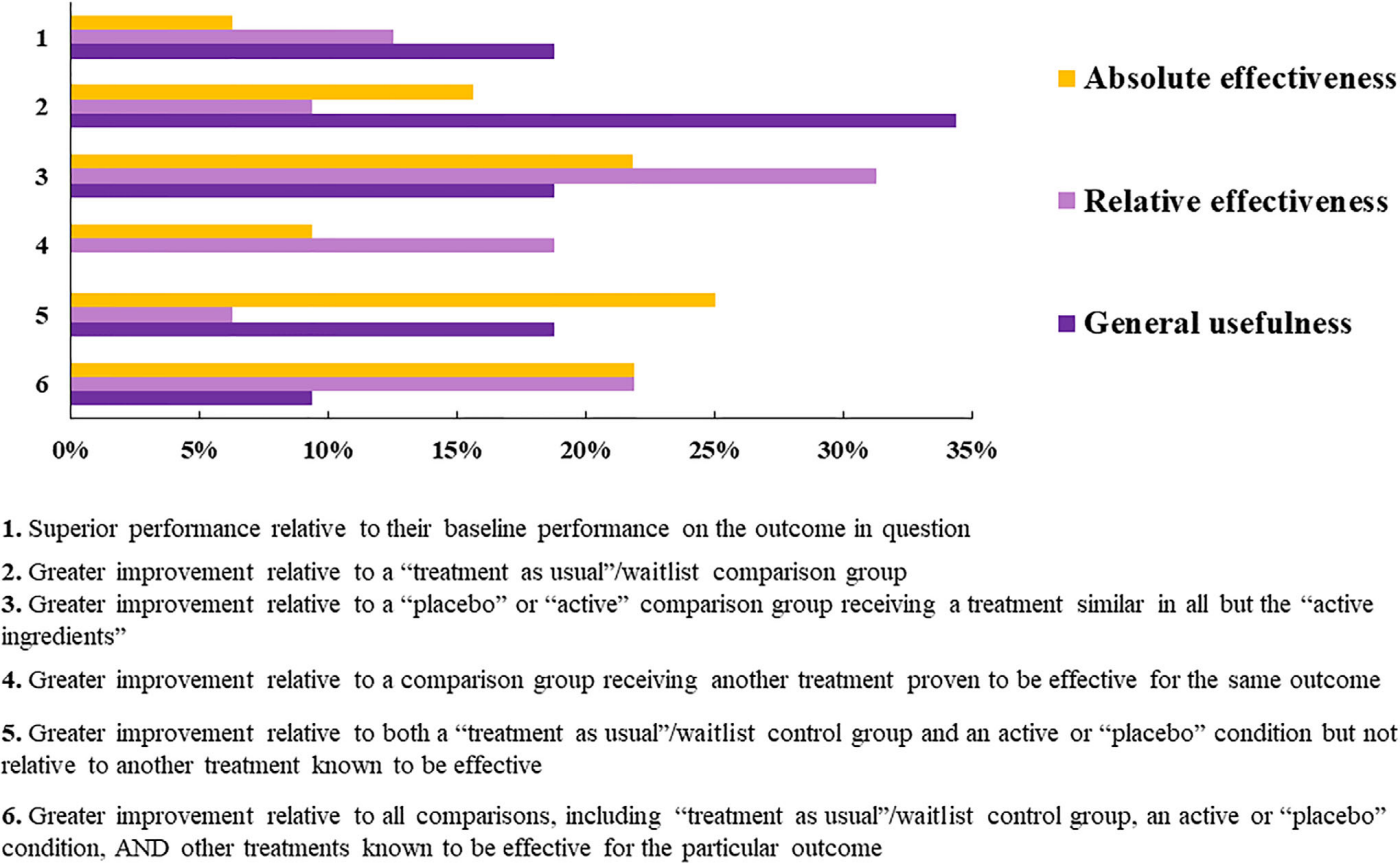
Assessment of efficacy

In addition, respondents were asked to consider the level of evidence in order to be convinced of the *general usefulness*, and *relative* and *absolute effectiveness* of COTs (Figure 8). There was less agreement among respondents regarding the level of evidence necessary for absolute effectiveness than for general usefulness and relative effectiveness of COTs. Briefly, for general usefulness of COTs, the main criteria deemed was *Greater improvement relative to a treatment as usual/waitlist comparison group*. For relative effectiveness, the main choice was *Greater improvement relative to a placebo or active comparison group receiving a treatment similar in all but the active ingredients*. And regarding the absolute effectiveness, the main choice was *Greater improvement relative to both a treatment as usual/waitlist control group and an active or placebo condition but not relative to another treatment known to be effective*.

**FIGURE 9:**
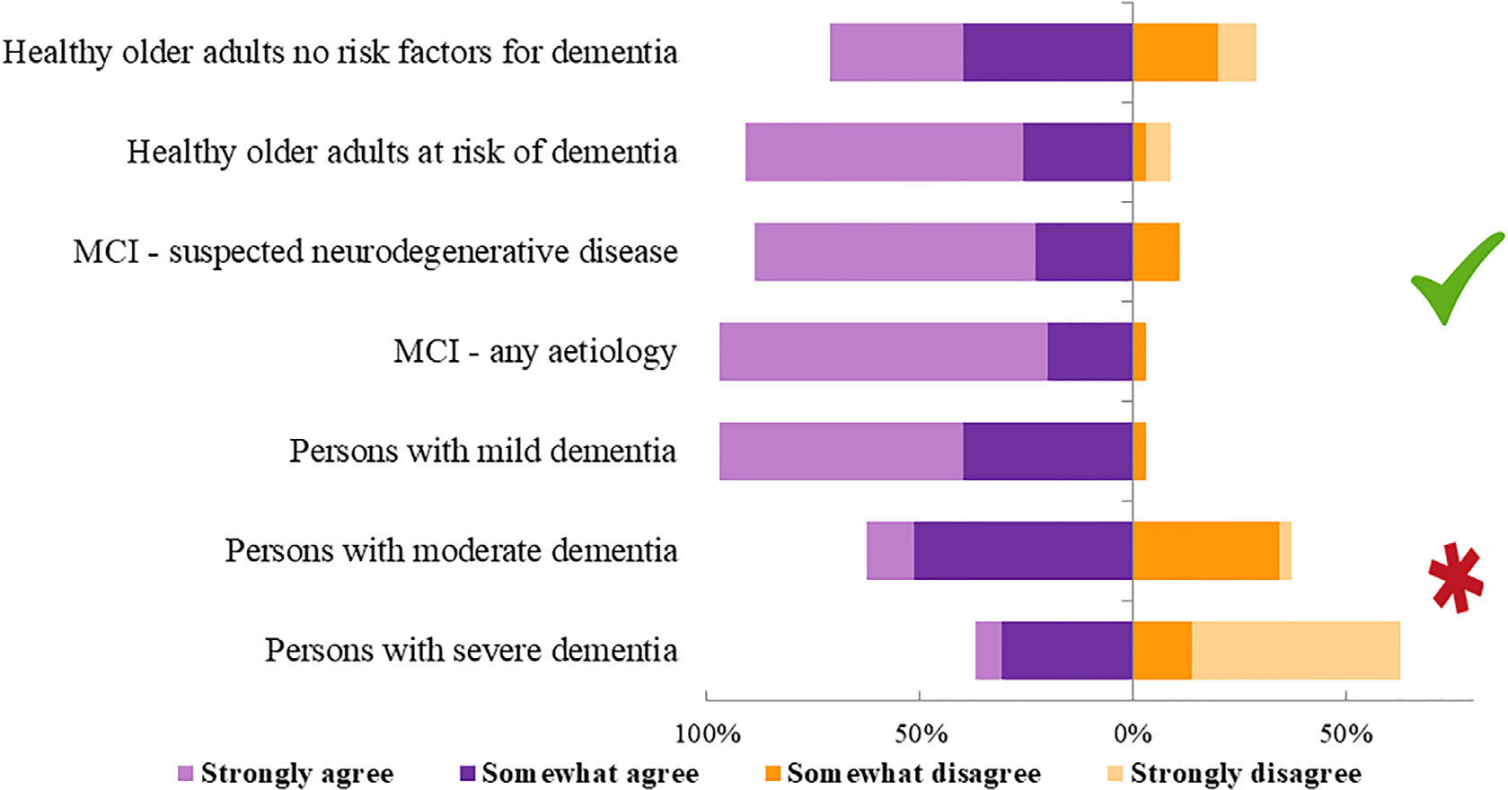
COT recommendations: in which population should be offered?

Finally, we observed a clear agreement among respondents that COTs should be offered to CU older adults (with and without risk of dementia), MCI, and mild dementia, and not much for those with moderate and severe dementia (Figure 9). In terms of prescription of COTs, most responders (56.2%) believed that *evidence of some usefulness in relation to cognitive functioning and/or a clinically meaningful outcome* is enough to recommend COTs, while 25% believe that is necessary to establish *absolute efficacy* in order to prescribe COTs. Finally, 50% of respondents believed that there is enough evidence that COTs can prevent dementia, indicating a lack of consensus for the role of COTs in dementia prevention.

## 4 DISCUSSION

The current study summarized the beliefs, knowledge, and practices of 39 experts in the field of COTs in older adults who responded to our survey invitation. We identified several areas of relative agreement among respondents, as well as some areas of relative disagreement, and these are briefly summarized and discussed below.

### 4.1 Key intervention features

There was relative consensus on the general features essential for COTs success, including that (1) the intervention needs to be adaptive in difficulty; (2) participants need to be given the opportunity to *identify* and *resolve* barriers to *adherence* and *performance*; (3) and participants need to be given *practical* and *emotional support*. Nevertheless, these key ingredients are not always formally incorporated, examined/monitored, or reported. For instance, a meta‐analysis identified that few COT studies reported measurements of adherence,^28^ and there is evidence that unsupervised interventions (ie, with less support) are less effective than supervised ones.^17^ In addition, an adaptive nature of COTs and tailoring to the needs of an individual may play a major role in motivation, adherence, and clinical significance of the results. Although adaptive computerized COTs have shown additional benefits than no‐adaptive control protocols,^44^, ^46^, ^61^, ^67^ this is not always observed,^68^, ^69^ indicating the need of further research on this matter. For research into COTs to advance, it is not only essential that key common ingredients are routinely incorporated into interventions, but also that these components, including their dosing parameters, are clearly and accurately described in treatment protocols.

### 4.2 Approaches

In keeping with proposed classifications,^2^, ^8^, ^10^ there was a consensus that the main terms used to describe COTs (ie, *cognitive stimulation, training, and rehabilitation*) indeed reflect different treatment approaches with distinct mechanisms of action. It is notable that although emerging evidence from the neuroimaging literature points to distinct neural signatures underlying strategy‐based and rehearsal‐based COTs,^27^ the evidence to date is insufficient to clearly differentiate the broad approaches described in the literature in terms of underlying neurobiology.

### 4.3 Targets

We found relative consensus that COTs should target multiple cognitive and non‐cognitive outcomes. This belief is not in line with the evidence that single‐domain COT interventions are also associated with benefits. For instance, benefits are reported in COTs focused in WM or executive control for CU older adults,^9^, ^37^, ^40^, ^42^, ^43^, ^44^, ^70^, ^71^ and in episodic memory for amnestic MCI.^51^, ^52^, ^54^ Likewise, a meta‐analysis on COTs in MCI found a significant overall effect for intervention content, indicating that memory‐focused intervention was more effective than multidomain approach, although the latter also showed a significant effect on cognitive performance.^28^

We found relative agreement that COTs should target impaired or weak cognitive functions rather than intact cognitive functions in MCI and dementia. COTs targeting cognitive weakness are frequently observed in people with MCI (eg, episodic memory), which show an improvement in memory performance following COTs.^10^, ^24^, ^26^, ^28^, ^54^ Studies of multi‐domain cognitive training, and in which both intact and impaired cognitive domains are likely to receive some training, have also been associated with cognitive benefits.^26^, ^28^, ^61^ Conversely, PwD may show more limited cognitive improvements following COTs,^2^, ^26^, ^31^ possibly due to more severe impairments in multiple cognitive domains. Hence, for PwD (and to a lesser extent, those with MCI) to benefit from a COT, and particularly to improve new learning, it may be essential to make use of relatively preserved skills and abilities, such as procedural learning.^36^, ^72^ For instance, the errorless learning technique can optimize learning by using feed‐forward instructions in order to prevent people from making mistakes during the learning process.^36^ Likewise, a goal‐oriented rehabilitation approach also builds on relatively preserved skills in order to facilitate learning and this approach has shown benefits in terms of everyday functioning.^33^

### 4.4 Strategies and techniques

Experts seem to agree that cognitive strategies should be incorporated into COTs when the target population is CU and MCI, but no consensus was found concerning people with dementia. This is in line with work showing that mnemonic strategies can improve cognitive performance in CU or MCI population, such as method of loci,^9^, ^24^, ^50^ story making, semantic association or clustering,^24^, ^49^ PQRST,^24^, ^50^ calendar/notes system,^57^ visual imagery,^24^, ^50^, ^73^, ^74^ which has been also integrated into associative memory training for face‐name,^54^, ^75^ and object‐location.^51^, ^53^ Nevertheless, some techniques that facilitate implicit learning and skill acquisition have shown to be beneficial to individuals with more pronounced cognitive deficits (ie, MCI and dementia), such as errorless learning,^36^, ^74^, ^76^, ^77^ spaced retrieval,^72^, ^74^, ^78^ and vanishing cues.^77^, ^79^

### 4.5 COT design: format, setting, dose, and frequency

In terms of intervention format, there was an agreement that small group may be the optimal preference for CU and MCI. This belief likely reflects the view that social engagement is beneficial and therefore should be incorporated into COTs, in line with the evidence that social relations are protective for age‐related cognitive decline.^80^, ^81^, ^82^ In addition, it is likely that including the social component in COTs plays a role in motivation and adherence, and may also be more cost‐beneficial than an individual approach. Nevertheless, the individual format may facilitate the use of some technologies (eg, *apps* and virtual reality), and adapt the training difficulty, which has shown to be beneficial.^44^, ^61^, ^67^ More efforts should be done to incorporate both formats in the same protocol,^48^ which may result in additional benefits. For PwD, a relative consensus indicated a preference for a *one‐on‐one approach*, which allows tailoring of the intervention to the needs/goals of the individual, facilitates learning, and collaboration between the therapist, patient, and family/caregiver, consistent with the evidence from the goal‐oriented cognitive rehabilitation approach.^31^, ^33^

Experts regarded the combination of home, and community, or clinic as the optimal setting for interventions for people with MCI and dementia, although the literature focuses on one or other setting for the most part. In the case of people with MCI, there are several protocols delivered at home (eg,^61^; for reviews, see Hills et al.,^26^ or at clinic (for reviews see 10, 28), and an increasing number of studies combining clinic setting with home or structured homework.^24^, ^48^, ^57^, ^60^, ^74^, For PwD, there are several cognitive training protocols delivered in the community or residential care settings that have shown small to moderate effects when compared to a control condition, but no effects were found when compared with an alternative treatment.^2^ In addition, goal‐oriented cognitive rehabilitation tends to combine different settings when emphasizing the collaboration between therapist, patient, and family or caregiver, and this has been shown to be of benefit.^33^ Whether there are additional benefits from combining settings, and what is the optimal way to achieve this remains unclear. Regarding normal aging, it appears that experts believed that the COTs setting does not matter. Although this perception seems in line with the growing body of literature of home‐based computerized protocols for CU older adults,^11^, ^16^, ^17^ it contradicts the evidence that home‐based COTs are less effective than clinical‐based ones.^17^ Despite that, home‐based COTs tend to be more cost‐effective in comparison to therapist‐led, and have the potential advantage of being adaptive and scalable, allowing access to those who may be frail, have mobility limitations,^17^, ^84^ or reside in rural regions.

One of the great challenges is how *dose* is defined and measured in COTs. Dose can be broadly defined as the quantity of a therapeutic agent to be taken to achieve a specific effect. In the context of COTs, dose would refer to the exposure necessary to different factors, such as practice a determined cognitive process, or learn to use a strategy and an information.^10^ In the survey, most experts stated that *number of sessions per week* was the optimal way to define and measure dose in COTs, followed by *total amount of (training) time*. Although these dosing parameters are in wide use in COT protocols, they may not provide specific information on dose‐response relationships, since they do not directly show how much practice a participant *actually received* in a determined cognitive process or in learning a strategy. Specifically, the contents of a session can vary dramatically across participants even when a manualized intervention is used given the myriad of participant‐specific factors that can affect progress (eg, perseveration, set loss, inattention, fatigue). It is important that future studies attempt to provide more accurate information in relation to this matter, for instance using a *trial‐based approach*,^10^ or *gains per session*. It is worth noting that meta‐analytic studies did not find an effect of total intervention duration on COTs efficacy,^17^, ^19^ although qualitatively interventions that lasted 20 hours or more had larger effects than those that lasted <20 hours.^19^

Regarding frequency, there was an agreement that 1 to 2 times a week is optimal for CU, as reported previously.^3^, ^24^, ^45^, ^49^, ^50^, ^51^, ^53^ In addition, two meta‐analysis found that fewer weekly sessions (eg, 1‐2 or 1‐3 sessions) may be more effective than 4 to 5 sessions.^17^, ^19^ Nonetheless, evidence from individual studies shows that COTs delivered 3 times or more per week are associated with cognitive benefits.^40^, ^42^, ^43^, ^44^, ^46^, ^48^, ^67^ Concerning people with MCI, there was relative disagreement regarding the optimal treatment frequency, which may reflect that benefits are described in COTs incorporating sessions 1 to 2 times a week (for a review see^28^; and for more recent reports see,^24^, ^54^ 2019,^53^ but also 3 to 5 sessions per week.^60^, ^61^, ^85^, ^86^

### 4.6 Outcomes and measures

We observed a clear consensus that COTs for MCI should incorporate the measurement of subjective everyday/functional cognitive outcomes (informant or self‐reported). Nevertheless, studies do not routinely incorporate these types of measures, which may be important for evaluating transfer effects in daily activities. For instance, a randomized‐controlled trial (RCT) focused on the implementation of the use of calendar/notebook system found an improvement on activities of daily living measured by an informant‐based questionnaire.^57^ Likewise, functional status improved after a cognitive strategy training in measures assessing medication management and bill paying,^87^ and after strategy training in combination with education on lifestyle and psychosocial support.^73^ Other RCTs that have focused on mnemonic strategy training (MST) found increase in a self‐report measure of strategy use,^24^ reduction of cognitive complaints,^24^, ^88^ and frequency of memory mistakes in everyday life.^54^, ^89^ It is relevant to highlight that other COTs found benefits on cognitive performance but no changes in self‐report everyday life activities ^61^, ^74^.

There was agreement that both structured cognitive tasks and daily activities should be targeted in COTs for CU and MCI. However, structured cognitive tasks are more frequently incorporated in COTs for these populations (for reviews see^28^; Hill et al., 2016.^10^, ^16^, ^17^, ^19^, ^30^. Despite that, COTs targeting everyday life have been developed specifically for MCI.^25^, ^60^ In addition, efforts have been made to develop cognitive tasks that reflect real‐life difficulties, such as forgetting people's names or location of objects,^24^, ^51^, ^54^, ^75^ using a note system,^57^ or creating virtual reality environment simulating a real‐life situation (eg, supermarket).^90^, ^91^ Future studies should better combine these cognitive tasks and daily activities in order to enhance transfer effects to meaningful real‐life situations.

### 4.7 Transfer

Most responders believed that training cognitive strategies is critical to induce transfer of gains from trained to untrained tasks. Although strategy training protocols showed transfer effects from trained to untrained tasks^24^, ^50^, ^51^, ^53^, ^54^, ^55^ this is not consistent.^58^, ^59^ Likewise, some transfer effects have been shown in rehearsal approaches as well^11^, ^13^, ^16^, ^17^, ^19^, ^28^; therefore transfer effects are not exclusive from COTs focused on strategy training. It is hypothesized that when an individual acquires a new strategy to learn information, or to complete a task, they are likely to use it in different situations, enhancing transfer to different contexts. Although this seems a critical mechanism, there is not enough evidence that this is the main factor to contribute to transfer. Other factors such as dose may play a relevant role as well, as shown in dose‐response studies.^43^, ^92^ In addition, ecological training protocols and outcome measures may enhance transfer by creating a more daily‐life environment. It is critical that future studies address specific factors that contribute to context and content transfer.

### 4.8 Population benefit, prescription, and dementia prevention

There was relative agreement that people with MCI were more likely to benefit from COTs than CU, which may reflect a perception that improving cognition and function in clinical populations is particularly meaningful. This assumption is, however, not always supported by empirical evidence from studies that directly compared these populations. For instance, two studies did not find evidence that MCI or CU benefit differently from MST.^50^, ^51^ In contrast, others found that people with MCI improved more than CU following training of speed of information processing^93^ and MST,^58^ and conversely, that CU presented greater improvement after WM training.^86^ In addition, there is evidence that CU with better cognitive baseline would present larger training effects.^47^ Despite these findings, a meta‐analysis on COTs comparing different population did not find that MCI or CU individuals would benefit differently from COTs.^19^ Although it is to be expected that CU individuals would outperform people with MCI on both baseline and post‐intervention assessments, whether one group shows greater improvement following training relative to the other remains unclear. Regarding PwD, the survey indicated a clear consensus that PwD benefit less from COTs than people with MCI or CU, in line with the frequent negative or limited findings despite some cognitive benefits^2^, ^26^, ^31^ and functional improvements.^33^, ^34^

In terms of maintenance of training effects, experts agreed that both CU older adults and MCI might retain COT‐related gains in the long run (ie, over a year), although some benefits are likely to wane in the short term (weeks to 3 to 6 weeks). This perception is consistent with the evidence from ACTIVE, the cognitive training trial with the longest follow‐up period to date, which showed that COTs can attenuate cognitive and functional decline after 10 years,^3^ as well as reduce dementia risk.^5^ However, long‐term benefits from COTs in people with MCI is not frequently investigated and evidence beyond 1 to 2 years is limited.^59^ Nonetheless, there is consistent evidence that part of the benefits persist following relatively short term delays (ie, 1 to 6 months).^24^, ^51^, ^54^, ^61^, ^75^ A critical aspect for future studies using long‐term follow‐up (eg,>1 year) is how to interpret long‐term benefits considering that individuals with MCI often present with an underlying neurodegenerative disease and are therefore expected to deteriorate. Although there are encouraging data on cognitive benefits of COTs to older adults, there is little evidence (except for ACTIVE trail) on the effect of COTs on dementia risk.^5^ To understand the role of COTs on dementia prevention, future studies should provide more data on long‐term outcomes of COTs, such as incidence of dementia.

In conclusion, despite the heterogeneity in COTs and methodological limitations in the field, there are clearly several areas of agreements among clinical and research experts on critical features of COTs, and on study design and outcome measures. Nevertheless, expert opinions are not always supported by incontestable scientific evidence, suggesting that methodologic improvements are needed to provide high‐quality evidence, and to design, implement, and report COTs. These improvements may be facilitated by future development of guidelines for COTs research. There is a clear consensus that COTs provide benefits and should be offered to CU older adults (with or without risk factors for dementia), MCI, and mild dementia, but opinions differ for moderate and severe dementia. Despite the encouraging benefits of COTs for older adults, there is still no consensus on the potential role these treatments could play in relation to dementia prevention, indicating that future research should prioritize this aspect in order to better recommend COTs and potentially enhance dementia prevention worldwide.

## CONFLICTS OF INTEREST

The authors declare no conflict of interest
